# National Helpline for Problem Gambling: A Profile of Its Users' Characteristics

**DOI:** 10.1155/2015/659731

**Published:** 2015-05-03

**Authors:** Luca Bastiani, Maurizio Fea, Roberta Potente, Claudia Luppi, Fabio Lucchini, Sabrina Molinaro

**Affiliations:** ^1^Institute of Clinical Physiology, National Council of Research, Via Moruzzi 1, 56124 Pisa, Italy; ^2^Gioca Responsabile, FeDerSerD, Via Giotto 3, 20145 Milano, Italy

## Abstract

Gambling has seen a significant increase in Italy in the last 10 years and has rapidly become a public health issue, and for these reasons the first National Helpline for Problem Gambling (GR-Helpline) has been established. The aims of this study are to describe the GR-Helpline users' characteristics and to compare the prevalence rates of the users with those of moderate-risk/problematic gamblers obtained from the national survey (IPSAD 2010-2011). Statistical analysis was performed on data obtained from the counselling sessions (phone/e-mail/chat) carried out on 5,805 users (57.5% gamblers; 42.5% families/friends). This confirms that the problems related to gambling concern not only the gamblers but also their families and friends. Significant differences were found between gamblers and families/friends involving gender (74% of gamblers were male; 76.9% of families/friends were female), as well as age-classes and geographical area. Female gamblers had a higher mean age (47.3 versus 40.2 years) and preferred nonstrategy-based games. Prevalence rates of GR-Helpline users and of moderate risk/problematic gamblers were correlated (Rho = 0.58; *p* = 0.0113). The results highlight the fact that remote access to counselling can be an effective means of promoting treatment for problem gamblers who do not otherwise appeal directly for services.

## 1. Introduction

Opportunities for access to gaming venues have increased greatly. The wide availability of legalized games has led many to consider gambling to be a common social activity that is both legalized and a socially acceptable form of leisure activity.

Many studies have suggested that the high level of accessibility to legal gambling opportunities is associated with an increasingly prevalent participation and the appearance of disordered gambling [1–3].

A recent analysis by Williams et al. [4] concluded that the standardized past-year rate of problem gambling among the adult general population ranges from 0.5% to 7.6% (depending on the country and the survey year), with the average rate across all countries being 2.3%. These researchers estimated that the lowest standardized prevalence rates of problem gambling occur in Europe, more specifically Denmark, Netherlands, and Germany, with intermediate rates in North America and Australia, and also Sweden, Switzerland, Estonia, Finland, and Italy. The highest rates are observed in Asia and South Africa.

In Italy, a survey on prevalence in the general population (Italian Population Survey on Alcohol and Other Drugs—IPSAD), in 2007-2008, revealed that 42.1% of 15–64-year-olds had gambled during the previous 12 months and, of these, 5.2% were moderate-risk/problematic gamblers [5]. In the subsequent survey (IPSAD 2010-2011), prevalence of moderate-risk/problematic gamblers did not substantially change (5.6%) [6]. In recent years the Italian public gaming industry has been expanding very rapidly, collecting 54.4 billion euros in 2009, 61.4 billion in 2010, and 79.9 billion in 2011 [7], which, respectively, accounts for 3.7%, 4%, and 5% of the Italian GDP.

Researchers have suggested that the young people (i.e., younger than 29 years old) represent a segment more susceptible to gambling problems than adults [8]. Additionally, over the last decade, they have become the older adults [9–11]. In general the fast growing population is aged 60+ in almost all regions of the world, especially in the more developed countries [12] where greater life expectancies are observed.

Overall, gambling behaviors, both normal and problematic, are associated with males, whose rates are much higher than women (e.g., [4, 13, 14]). Some studies have shown that the average age of female problem gamblers is higher than males and that females progress towards gambling disorders faster [15–17]. This also associates with more severe psychiatric symptoms [18]. In the general population prevalence studies reveal a strong association between problem gambling and specific forms of gambling, for example, lottery, cards and bingo [19], casino table games, and electronic gaming machines [13, 20].

Overall, most people gamble recreationally, considering it a harmless form of entertainment. For some gamblers, however, this behavior becomes problematic and pathological, with adverse consequences for the gamblers, their families, and the community, involving impairment or loss of social relationships and financial resources, work and legal difficulties, and even physical health problems and antisocial behavior [21, 22]. It is estimated that, for every individual who develops a gambling problem, from 5 to 10 additional people (i.e., friends, family, and employers) are adversely affected [1].

The individual, social, and economic costs associated with gambling behaviors are so large that gambling and its related problems have become a significant and growing public health issue [3, 17, 23, 24]. This situation requires the development and implementation of both new and alternative approaches to treatment targeting a much broader range of problem gamblers. Unfortunately, only a small proportion of the individuals with gambling disorders (<10%) seek formal treatment in clinic based programs [22, 25].

The resistance to seek treatment for problem gamblers includes several personal factors (perceived stigma or shame, embarrassment/pride, difficulty in acknowledging the problem and denial, wariness about seeking professional help, or believing that they can handle the problem without external help) [26], but also some external barriers (lack of awareness of services, ignorance of the availability of treatment, geographical distance, existing time commitments, and work and domestic demands) [27].

Helpline services and online counselling (e.g., telephone, e-mail, and chat) could serve as alternative forms of access to treatment for gamblers who reject more traditional options. These interventions are less expensive than formal treatment [2] and can serve wide geographical areas, thus overcoming many barriers, increasing accessibility to professional treatment, and thus attracting new treatment candidates [9, 28].

Some studies have shown several concerns using online treatment support service, namely, guaranteeing privacy and anonymity, emotionally safe environment, convenience and issues regarding time, confidentiality, and flexibility [2, 29, 30].

Out of concern about severe adverse psychosocial consequences and prevalence rates of gamblers and problem gamblers, the Italian Federation of Workers of the Departments and Services Addiction (FeDerSerD) initiated and managed the first helpline and website service GIOCARESPONSABILE (GR-Helpline). This operates under the patronage of the Presidency of the Council of Ministers and is funded by GTECH Group (Lottomatica Group, Italy). The service, operating 13 h by phone and 24 h by web (e-mail and chat) each day, is friendly, confidential, convenient, freely accessible, and anonymous. It is managed by therapists and other professionals (e.g., psychologists, psychotherapists, and lawyers) who provide counseling and assistance to people who seek help for gambling problems reaching not just gamblers but also families, friends, and others indirectly involved.

Briefly, the service GR-Helpline consists of helpline and website, accessible daily between 9 a.m. and 10 p.m., from landline and mobile phone to the toll-free number (800 921 121) or online through the portal http://www.giocaresponsabile.it/, by chat and e-mail.

To promote awareness of this service, information leaflets were distributed to general practitioners and social and health services providers, besides the gambling venues themselves: the most important source of information appeared to be the network of general practitioners, followed by the web and the gaming establishments.

The anonymity of users is guaranteed by assigning a system-generated alphanumeric code: this enables deidentified surveys to collect information on gambling behavior and related problems. Moreover, this code enables retrieval of personal information and subsequent addition of information collected through further contact.

The team of psychologists, who have the initial contact with the caller, is supported by additional counselors (e.g., psychiatrists, psychotherapists, and lawyers) who can be activated upon caller request or by the team for issues that require more in-depth analysis. To better assist users, the gambling helpline also provides access to face-to-face counseling, by sending an e-mail containing the code of the users to the services that have joined the network GR-Helpline [31] and referred to the data base of the site. Similarly, care services that receive communication confirm whether or not the person is receiving treatment to provide a partial measure of effectiveness.

The main aims of the current study were

- to examine the characteristics of users to the gambling helpline, distinguishing between gamblers and those involved indirectly in gambling behavior (families/friends);
- to survey the characteristics of gamblers stratified by different age classes and by different forms of games;
- to compare the prevalence rates of users with the findings that have emerged from a national survey.

## 2. Methods

### 2.1. Data Collection

Data were obtained from recorded calls to GR-Helpline received between November 2009 and November 2012 and only involving gamblers and their families and/or friends. This excluded accesses by other users, for example, members of the professional community.

Online counselling is conducted using an interview based on the diagnostic protocol of Ladouceur and colleagues [32]. It is a semistructured clinical interview based on DSM-IV criteria and composed of 26 questions on pathological gambling. In addition to the diagnostic criteria described in the DSM-IV, the interview investigates other aspects such as the reasons for the consultation, the events that have led to the decision, information on how you obtained your gambling habits, details of the gambling problem, and the presence of other addictions.

The psychologists, who conduct counselling via telephone and via web, perform an assessment of severity and enter in the data base only those subjects whose conditions can be considered problematic. As an additional criterion for validation, only calls via chat and phone that lasted more than 2 and 7 min, respectively, are considered valid.

The counseling sessions for gambling behavior considered valid were 11,113: 71.1% by phone, 22.4% by e-mail, and 6.5% via online chat; for 5,805 users, 3,337 were gamblers and 2,468 families/friends. Families/friends, compared to gamblers, preferred accessing the GR-helpline by phone (81.7% versus 64.0%), while gamblers preferred using e-mail (28.5% versus 13.1%).

The variables selected for the present analysis include

- demographic characteristics: gender, age, and geographical region of residence;
- source of information on service: public health services, media, gaming environments, or other places;
- forms of gambling, as defined in other studies [33]:
  - strategy-based games: poker, blackjack, horse racing, sports, and other betting,
  - non-strategy-based games: lottery, video lotteries and new slot machines, pull tabs, bingo, and keno,
  - both;
- gambling behaviour:
  - frequency: regular (up to 6 times a week); moderate (1-2 times a week); occasional (less than once a week),
  - amount of money spent weekly: <1,000 euros; from 1,000 to 10,000 euros; >10,000 euros,
  - amount of money lost: <1,000 euros; from 1,001 to 10,000 euros; >10,000 euros,
  - presence of indebtedness (yes/no).

Other data for this study were drawn from IPSAD 2010-2011, a survey on the Italian population between 15 and 64 years old, and concerning the classification on gambling using the Canadian Problem Gambling Index (CPGI) Short form scale [6, 34–36]. The CPGI consists of 9 questions that are scored on a four-point Likert scale. The response categories are 0 = never, 1 = sometimes, 2 = most of the time, and 3 = almost always. A composite score equaling 0 identifies no problem gambling, 1-2 low problem gambling, 3–7 moderate problem gambling, and 8–27 severe problem gambling. The psychometric properties of CPGI investigated in the Italian validation study [6] showed high reliability (Cronbach's alpha = 0.87).

### 2.2. Statistical Analysis

Analyses were performed using the statistical package SPSS (version 20).

Users were divided into two groups: gamblers (individuals directly exhibiting gambling behavior) and families/friends. Users' demographic characteristics were summarized for each of the two groups, using percentages; and comparing between groups was done using the Chi-square tests.

Gambling behaviour was analysed across the three different gambling forms (strategy-based; non-strategy-based; both), stratified by age groups (15–24 years, 25–44 years, 45–64 years, and ≥65 years), and the Chi-square test was performed comparing each variable across the three groups.

Finally, to compare the characteristics of GR-Helpline users with those of moderate-risk/problematic gamblers obtained from the IPSAD 2010-2011 survey, we also used age stratification of 15–24 years and 25–64 years.

Spearman's rho correlation analysis was evaluated to compare the regional prevalence of moderate-risk/problematic gamblers (IPSAD 2010-2011 survey) with those of regional users who seek help.

## 3. Results

### 3.1. Characteristics of Users

The analyzed sample included 5,805 users: 57.5% were gamblers and 42.5% were families/friends.

Table 1 shows the demographic user characteristics describing gamblers and families/friends separately.

Male gamblers were more frequent users than females (ratio for males/females is 3 : 1) and had a lower mean age (40.2 versus 47.3 years), while female families/friends were more frequent users than males (ratio for males/females = 1 : 3).

The prevalence of gamblers and families/friends was greater (52.2% and 50.2%, resp.) from regions in northern Italy than those from southern and central Italy.

### 3.2. Gambler User and Gambling Behaviour Characteristics

Most of the gamblers preferred non-strategy-based games (77.4%; strategy-based games: 11.4%; both: 11.2%), increasing with increased age classes. Non-strategy-based games were preferred by females (m = 74.1%; f = 87.6%); a significant difference in gender was found within the 25–64-year-olds (*p* < 0.05).  Table 2 presents the bivariate analysis of some aspects related to problem gambling behavior comparing the three gambling forms stratified by age classes.

The percentage of those exhibiting a regular frequency of weekly gambling was high for all four age classes and the gambling forms.

Although in all age classes the most common category of weekly expenditure is <€1,000, compared to others, young strategic gamblers have the highest rate of spending in the category >€10,000 (5.9%).

Among young people, and in all gambling forms, the highest percentage of amount of money lost is in the category of €1,000–10,000. In all other age classes the gambler users claim to have lost >€10,000.

Indebtedness reported by the youngest age classes is distributed similarly across different gambling forms.

Between gamblers aged 25–64 years, the strategy-based gamblers reported having the greatest debt. In the older group, those who gamble both in strategy-based and non-strategy-based games have the highest percentage of those who have contracted debts.

Table 3 shows the results of the Chi-square test analysis of sociodemographic characteristics comparing the two age classes in gambler users of the GR-Helpline and moderate-risk and problematic gamblers detected through the CPGI, included in the IPSAD 2010-2011 survey. Statistically significant differences were detected between genders compared in young adult groups but not in higher age classes. For both age classes, significant differences were exhibited regarding employment status and gambling forms.

### 3.3. Comparison between Prevalence Rates of Users and Moderate-Risk/Problematic Gamblers

**Figure 1:**
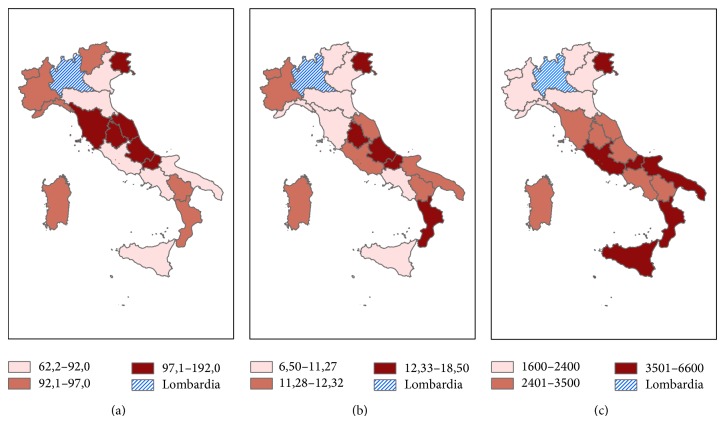
Regional distribution of informative materials of GR-Helpline (a); regional distribution of gamblers and families/friends using GR-Helpline (b); regional distribution of moderate-risk/problematic gamblers of IPSAD 2010-2011 survey (c), expressed as rate 1/100.000 inhabitants aged 15–64 years.

Figure 1(a) shows the regional distribution rate of informative materials about the GR-Helpline sent to general practitioners. Compared with other Italian regions, Lombardy exhibits the highest coverage: in this region over 14,000 information leaflets were sent out, while in the other regions the average was 1,600. Therefore, Lombardy (where all general practitioners and other specialists received information leaflets) was excluded in the analysis.

Prevalence rates of gambler and families/friends users to GR-Helpline and of moderate-risk/problematic gamblers, as identified using the IPSAD 2010-2011 survey, were correlated, and Spearman's rho correlation coefficients estimate was 0.58 (*p* = 0.0113) (Figures 1(b) and 1(c)).

## 4. Discussion

This study is the first in the Italian population that explores the characteristics of the people who accessed, in the first 3 years of activity, the helpline and online service for problem gambling, GR-Helpline.

Our findings show that the use of counseling by telephone was more frequent than the other two online modalities (e-mail and chat), primarily for family members/friends and among middle-aged and older adults. This support, which is free and easily accessible and is more widespread and similar to face-to-face counselling, best meets the needs and habits of the middle-aged adults and the elderly, while younger people tend to also utilize chat and e-mail [28, 29]. As observed in other researches [9, 33], our study shows that it is possible to associate the preference for online contact modalities with the forms of gambling: among strategy-based gamblers, the forms of gambling (such as poker, blackjack, or betting), mainly practiced on the Internet, are more common in young and young adult gamblers.

Although there are more non-strategy-based gamblers than those who prefer strategy-based forms or who use both forms of gambling, our findings suggest that the strategy-based games are associated with a greater gambling frequency (up to 6 times a week) and spending more money each week. Higher losses and indebtedness are also found among middle-aged and older groups in concordance with other studies [9, 11].

As reported by Bellringer and colleagues [37], for users of the gambling helpline, the financial consequences of gambling losses are a principal cause for conflict and family problems: legal requests by family members to resolve debts and protect assets comprised 20% of requests for help received by GR-Helpline.

Another finding that should be highlighted concerns gender differences related to gambling behavior: women gamblers are, on average, older than male gamblers and more likely to report problems with non-strategy-based forms of gambling. As reported in the studies by Potenza and colleagues [15, 38], the different gambling patterns between genders suggest that women may engage in more escape-oriented forms of gambling (e.g., slot machine and table and instant lotteries) and men in more action-oriented forms, seeking to challenge themselves and their skills.

From analysis of these early years of helpline activity and from the initially observed data we can state that problem gambling extends beyond the gamblers themselves. About half of the users were significant others who sought help due to someone else's gambling behavior; these are largely female with a percentage similar to that of male gamblers. This finding was also observed by other gambling helplines (e.g., [39, 40]).

Although a correlation has been detected between the prevalence rates of users of GR-Helpline and moderate-risk/problematic gamblers, as the IPSAD 2010-2011 survey reported, it should be underlined that the studies investigated two different populations.

People who contact the helpline are therefore a self-selected population of individuals facing a problem and probably believe that they could personally deal with it, while the national survey IPSAD 2010-2011 estimates, in the general population, the existence of the problematic nature of gambling through a validated screening instrument (CPGI). The subjects identified in the survey show problematic profiles, but it is not obvious that they are aware of or want to report their problem [41]; therefore it would be appropriate to include questions within the surveys relating to previous or current requests for care, to correlate the prevalence data with trends in the expression of the demand for care.

As concerns the helpline, the request for help is also correlated to socioenvironmental variables (e.g., “what, where, and how” information is spread [24], “what the social perception of the problem is” [21, 37, 42], “what kind of policies are implemented regarding gambling,” and “how present and widespread the network of care and social welfare is” [43]).

As already recognized by many authors [2, 43, 44] it is important to provide opportunities for differentiated treatments [45], not only as to methods, types of care, and counseling [46], but also regarding settings [47], availability in time, and means of access. It may also be useful to provide information channels for different targets, not only in accordance with the type and mode of game but also with respect to age [48]. Remote access to counseling can be an effective instrument of promoting treatment for problem gamblers who do not otherwise appeal directly for services.

## 5. Limitations and Future Research

It is important to acknowledge several limitations. Although the study involved the use of a semistructured clinical interview based on DSM-IV criteria [32], interrater reliability among helpline staff was not assessed. We recognize the limits of the comparison between the gambling behaviors detected by a semistructured interview with the gamblers identified in the population study through a self-report instrument.

Our findings show that the prevalence rate of GR-Helpline older age group users is nonnegligible: in Italy there are no specific studies on subjects aged over 64 years, so in the last IPSAD survey the sampled population was extended up to 75-year-old subjects.

The findings could be useful for assessing the evolution of the phenomenon and for programming public health and social care policies.

## 6. Conclusion

The present study, describing the first experience of the National Helpline for Problem Gambling (GR-Helpline) in Italy, shows that gambling is a problem that not only concerns the gambler, but involves a significant impact on the family. The users of the GR-Helpline are, in fact, equally gamblers and families/friends in an approximately similar rate.

Remote access (e.g., telephone, e-mail, and chat) is an effective instrument to counselling and to access treatment for problem gamblers and their families as an alternative and more immediate and private form than conventional services. The study also highlights the fact that problem gambling concerns all age groups, even the elderly, to whom we should be paying more attention.

The spread of problem gambling in this age group is likely to be found also in the marketing strategies that increase the gaming opportunities.

The prevalence rates of gamblers and families/friends users (GR-Helpline) are correlated with prevalence rates of moderate-risk/problematic gamblers detected by the national survey (IPSAD 2010-2011), and this evidence should be taken into account in the development and planning of national and regional social health policies.

## Figures and Tables

**Table 1:** Characteristics of gamblers and families/friends using GR-Helpline.

Characteristics	Gamblers (*n* = 3,337)	Families/friends (*n* = 2,468)	*p* value
Gender			
Male	74.0%	23.1%	<0.05
Female	26.0%	76.9%	
Age			
15–24 years	7.6%	4.7%	<0.05
25–44 years	51.9%	47.2%	
45–64 years	36.0%	40.6%	
≥65 years	4.5%	7.5%	
Mean age			
Male	40.2 years (ds 12.0)	44.1 years (ds 14.3)	
Female	47.3 years (ds 12.6)	44.7 years (ds 12.8)	
Geographical area			
North	52.2%	50.2%	<0.05
Center	17.8%	16.5%	
South/islands	30.0%	33.3%	
Source of information			
Public health services	39.2%	53.7%	<0.05
Media (e.g., TV, web, or radio)	22.5%	24.8%	
Gambling venues	14.7%	5.6%	
Other modes	23.6%	15.9%	

**Table 2:** Characteristics of gamblers using GR-Helpline stratified by age classes and gambling form expressed as percentage.

	15–24 years				25–44 years				45–64 years				≥65 years			
Characteristics of gambling behavior	Strategy-based	Non-strategy-based	Both	*p* value	Strategy-based	Non-strategy-based	Both	*p* value	Strategy-based	Non-strategy-based	Both	*p* value	Strategy-based	Non-strategy-based	Both	*p* value
	(%)	(%)	(%)		(%)	(%)	(%)		(%)	(%)	(%)		(%)	(%)	(%)	
Frequency																
Regular	94.7	84.4	94.4	ns	83.7	79.8	79.3	ns	79.6	84.0	76.8	ns	80.0	86.4	100.0	ns
Moderate	5.3	8.9	0.0		7.3	13.8	12.4		14.8	10.1	14.6		20.0	6.8	0.0	
Occasional	0.0	6.7	5.6		8.9	6.4	8.3		5.6	5.9	8.5		0.0	6.8	0.0	
Amount of money spent weekly																
€1–1,000	94.1	94.9	94.7	ns	79.8	86.6	82.8	ns	78.9	84.2	85.3	<0.05	80.0	94.4	57.1	<0.05
€1,001–10,000	0.0	5.1	5.3		19.2	13.2	17.2		21.1	15.8	13.3		20.0	5.6	42.9	
€10,001 or more	5.9	0.0	0.0		1.0	0.1	0.0		0.0	0.0	1.3		/	/	/	
Amount of money lost																
€1–1,000	18.2	5.5	0.0	ns	4.7	3.1	0.0	<0.05	2.8	2.3	2.8	ns	0.0	3.4	0.0	ns
€1,001–10,000	63.6	43.6	41.7		27.1	28.1	17.0		8.3	25.6	18.3		0.0	34.5	0.0	
€10,001 or more	18.2	50.9	58.3		68.2	68.8	83.0		88.9	72.1	78.9		100.0	62.1	100.0	
Presence of gambling indebtedness																
No	88.6	82.3	88.5	ns	59.9	70.6	67.6	<0.05	57.4	68.1	55.7	<0.05	100.0	73.6	37.5	<0.05
Yes	11.4	17.7	11.5		40.1	29.4	32.4		42.6	31.9	44.3		0.0	26.4	62.5	

**Table 3:** Characteristics of gamblers using GR-Helpline and of moderate-risk/problematic gamblers classified by CPGI included in IPSAD 2010-2011 survey.

Characteristics	Gambler users GR-Helpline		Moderate-risk/problematic gamblers IPSAD 2010-2011		15–24 years	25–64 years
	15–24 years (%)	25–64 years (%)	15–24 years (%)	25–64 years (%)	*p* value	*p* value
Gender						
Men	92.0	74.7	70.9	78.6	<0.05	ns
Women	8.0	25.3	29.1	21.4		
Employment status						
Employed	61.4	74.0	19.6	62.3	<0.05	<0.05
Unemployed/not economically active	38.6	19.2	80.4	28.2		
Retired	0.0	6.8	0.0	9.4		
Gambling forms						
Strategy-based	20.1	11.0	39.6	7.7	<0.05	<0.05
Non-strategy-based	64.9	77.9	18.9	52.7		
Both	14.9	11.1	41.5	39.6		
